# The complex roles of space and environment in structuring functional, taxonomic and phylogenetic beta diversity of frogs in the Atlantic Forest

**DOI:** 10.1371/journal.pone.0196066

**Published:** 2018-04-19

**Authors:** Thiago A. Leão-Pires, Amom Mendes Luiz, Ricardo J. Sawaya

**Affiliations:** 1 Programa de Pós-Graduação em Ecologia, Instituto de Biologia, Universidade Estadual de Campinas (UNICAMP), Campinas, São Paulo, Brazil; 2 Centro de Ciências Naturais e Humanas, Universidade Federal do ABC, São Bernardo do Campo, São Paulo, Brazil, São Paulo, Brazil; National and Kapodistrian University of Athens, GREECE

## Abstract

Ecological communities are complex entities that can be maintained and structured by niche-based processes such as environmental conditions, and spatial processes such as dispersal. Thus, diversity patterns may be shaped simultaneously at different spatial scales by very distinct processes. Herein we assess whether and how functional, taxonomic, and phylogenetic beta diversities of frog tadpoles are explained by environmental and/or spatial predictors. We implemented a distance–based redundancy analysis to explore variation in components of beta diversity explained by pure environmental and pure spatial predictors, as well as their interactions, at both fine and broad spatial scales. Our results indicated important but complex roles of spatial and environmental predictors in structuring phylogenetic, taxonomic and functional beta diversities. The pure fine-scales spatial fraction was more important in structuring all beta diversity components, especially to functional and taxonomical spatial turnover. Environmental variables such as canopy cover and vegetation structure were important predictors of all components, but especially to functional and taxonomic beta diversity. We emphasize that distinct factors related to environment and space are affecting distinct components of beta diversity in different ways. Although weaker, phylogenetic beta diversity, which is structured more on biogeographical scales, and thus can be represented by spatially structured processes, was more related to broad spatial processes than other components. However, selected fine-scale spatial predictors denoted negative autocorrelation, which may be revealing the existence of differences in unmeasured habitat variables among samples. Although overall important, local environmental-based processes explained better functional and taxonomic beta diversity, as these diversity components carry an important ecological value. We highlight the importance of assessing different components of diversity patterns at different scales by spatially explicit models in order to improve our understanding of community structure and help to unravel the complex nature of biodiversity.

## Introduction

Why is biodiversity distributed non-randomly throughout space? What determines the structure and patterns of biological diversity in communities? These are some of the key questions proposed by prominent naturalists and ecologists, such as MacArthur and Levins [1] and Diamond [2], over the last 100 years. A myriad of questions involving the factors that influence the origin and distribution of biodiversity has since emerged. In order to properly address these questions, we must first understand how biodiversity can be measured and described. It is widely recognized that biodiversity can be decomposed into three key components: taxonomic, functional and phylogenetic diversities [3]. The first of these components, the classical measure of diversity at species level, results from species richness and relative abundance in a given community, without regards to evolutionary differences among species or other taxonomic levels (e.g. genus or families). The second component is the evolutionary history shared by species in a community, expressed as phylogenetic diversity [3,4]. The third component corresponds to the diversity of phenotypic traits of species in a community [5].

Spatial variation in species composition (i.e. species turnover), or simply β diversity emerges as the outcome of different processes occurring on distinct scales of space and time, and acting asymmetrically on an assemblage of communities. Niche theory states that local niche-based processes, such as environmental control (e.g., [6]), could determine the occurrence of species in communities. On the other hand, stochastic and neutral processes, including ecological drift and random dispersal, could also be involved in determining local community diversity and thus spatial variation among communities [7]. At broader scales, beta diversity could be a result of biogeographic and evolutionary processes, such as speciation, extinction and dispersal of lineages [8,9]. These processes can synergistically influence the regional species pool from which local communities are assembled [8,10]. Taxonomic Beta Diversity (**TBD**) represents spatial variation in community composition, but is usually considered not representative of the phylogenetic and functional differences among communities. Functional Beta Diversity (**FBD**) represents more of the ecological and biological association between organism traits and the environment, which is the core of niche-based processes [11,12]. Phylogenetic Beta Diversity (**PBD**) represents the role of evolutionary and biogeographic processes, such as speciation and dispersal, in structuring community diversity [4].

We could start investigating the processes related to community structure by understanding what predictors best explain the variation observed in biological diversity among assemblages. The predictors most commonly assessed correspond to a set of environmental variables that are related to species habitat use and phenotypic traits, which regulate niche overlapping of species and consequently determine their occurrence in a given assemblage [1,13]. In addition, spatial predictors have been employed to unravel their influence in the structure of communities, due to the spatial structure of neutral and niche based processes [14,15]. Spatial structure of communities can be the outcome of two principal sources and could not be unambiguously interpreted: environmental predictors that are themselves spatially structured, or spatial autocorrelation [7,16]. In the last few decades it has come to be acknowledged that patterns of diversity may be structured simultaneously by stochastic (neutral) and by niche-based processes at different spatial scales [16,17]. Thus, in order to understand how ecological communities are structured, it has become imperative to investigate, simultaneously, whether spatial and environmental predictors regulate diversity patterns of communities, and if they do so, then how.

Neotropical anurans are an excellent model for investigating the roles of ecological, evolutionary, and spatial processes in structuring ecological communities. In Brazil, they exhibit a high species diversity, with 1026 species recorded [18], and show both broad and narrow patterns of geographic distribution [19]. This variation in distributional patterns of anurans is widely considered to be a result of ecological, spatial and evolutionary mechanisms, such as environmental control and dispersal limitation [20,17]. It is assumed that these patterns can be explained by the extensive phenotypic and behavioural variability of the group, as well as their complex life cycle and permeable skin [21–23]. The occurrence of tadpoles in particular is recognized to be affected by habitat variables such as canopy cover, pond vegetation structure and pH [17,24,25].

Several studies have evaluated the mutual influence of ecological and spatial processes on community structure by testing which environmental and spatial predictors best explain patterns of taxonomic diversity of anurans (e.g. [17]). However, this framework has yet to be applied to other components of diversity, and especially with anuran communities (but see [26,17]). Furthermore, more integrated approaches have become increasingly important in understanding the origin and maintenance of beta diversity [9,17,26,27]. Herein we assess the relative influence of spatial and environmental predictors on functional, taxonomic and phylogenetic beta diversities of tadpoles in coastal plains of the Atlantic Forest in Southeastern Brazil. As stated above, **PBD** can represents a proxy to biogeographical and evolutionary processes, such as dispersal and speciation, which are spatially structured, so we expect that **PBD** will be more related to spatial predictors of communities. On the other hand, since **FBD** and **TBD** are more related to ecological processes, such as environmental control, they are expected to be better explained by environmental predictors.

## Material and methods

### Ethics statement

Collection permits were provided by Instituto Chico Mendes de Conservação da Biodiversidade (ICMBio) (#31554–1). Field studies did not involve endangered or protected species. Less than ten individuals per species were captured in accordance to the collection permits, killed using lidocaine, and preserved in 70% alcohol as vouchers, following the suggestion of McDiarmid [28] for amphibians. In 2010, beginning of the field sampling planning, there was no need for approval by any Institutional Animal Care and Use Committee (IACUC) or equivalent animal ethics committee in Brazil and our graduation program. All sampling procedures were reviewed and specifically approved as part of obtaining the field permits by ICMBio (see above) and Comissão Técnico-Científica do Instituto Florestal (COTEC; a committee of Instituto Florestal, a public research agency and owner of the reserves) (Processo SMA # 260108–002.279/2010).

### Study area and taxonomic data

**Fig 1:**
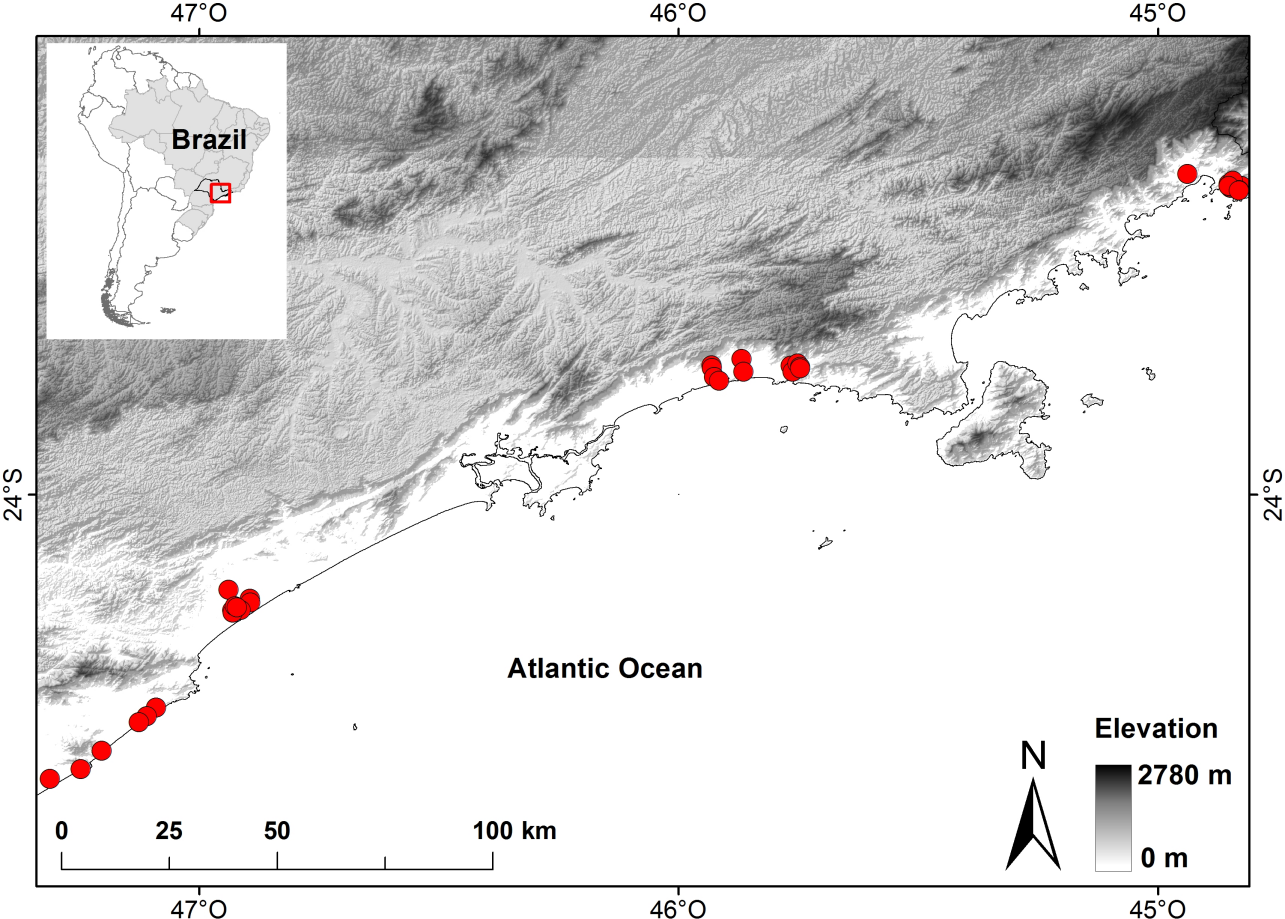
Study area and topographic complexity of Atlantic Forest of coastal plains in the state of São Paulo in Southeastern Brazil. Sampled ponds are represented by red circles (N = 37).

The study took place in the coastal plains of the state of São Paulo in Southeastern Brazil, and includes four natural geomorphological units, as proposed by ([29], see also 9) (Fig 1). The region encompasses about 550 km of coastal plains of Atlantic Forest bounded to the south and north by Precambrian basements of the Serra do Mar mountain range and divided by narrow headlands of Precambrian rocks [29,30]. The vegetation is composed of herbs, shrubs and forest formations classified as “restinga” forests and ombrophilous lowland forests within the Atlantic Forest domain [31]. Both of these formations are heterogeneous with regard to canopy height, hydric saturation of soil and distance from the slopes of the Serra do Mar (Fig 1).

We sampled 37 ponds distributed throughout the study area (Fig 1). Tadpoles and their potential fish predators were sampled in all suitable microhabitats available in the ponds for one hour during three different sampling surveys. Specimens were collected and identified to species level in the laboratory, and deposited in the “Coleção Científica de Anfíbios” of Universidade Estadual Paulista “Júlio de Mesquita Filho”, São José do Rio Preto, São Paulo, Brazil.

We constructed a presence–absence community composition matrix based on the species sampled in each pond. Since tadpole abundance in ponds is likely more related to species reproductive modes and strategies than to spatial or environmental influences [32,33], we used only species occurrence in communities in the taxonomic composition matrix.

### Environmental, phylogenetic and trait data

We measured the following environmental variables in each studied pond: area, water depth, diversity of internal and external vegetation structure, canopy cover, presence of potential predators (fish), pH, water temperature, water conductivity, and oxygen dissolved (see S1 Table and S3 Table, for further details). The proportion of canopy cover was measured using a Spherical Crown Densiometer. We transformed continuous variables (water depth, area, water temperature, water conductivity and oxygen dissolved) with Gower standardization as recommended by [34]. In this way, all numerical variables had the same weight in the analysis. Based on Pearson correlation (r < 0.60) variables were not considered correlated with each other, and then all environmental predictors were used to construct the models. We performed a Stepwise Model Selection based on adjusted R squared (R^2^adj), to select a set of environmental variables that best explain the variation in the components of beta diversity [35].

**Fig 2:**
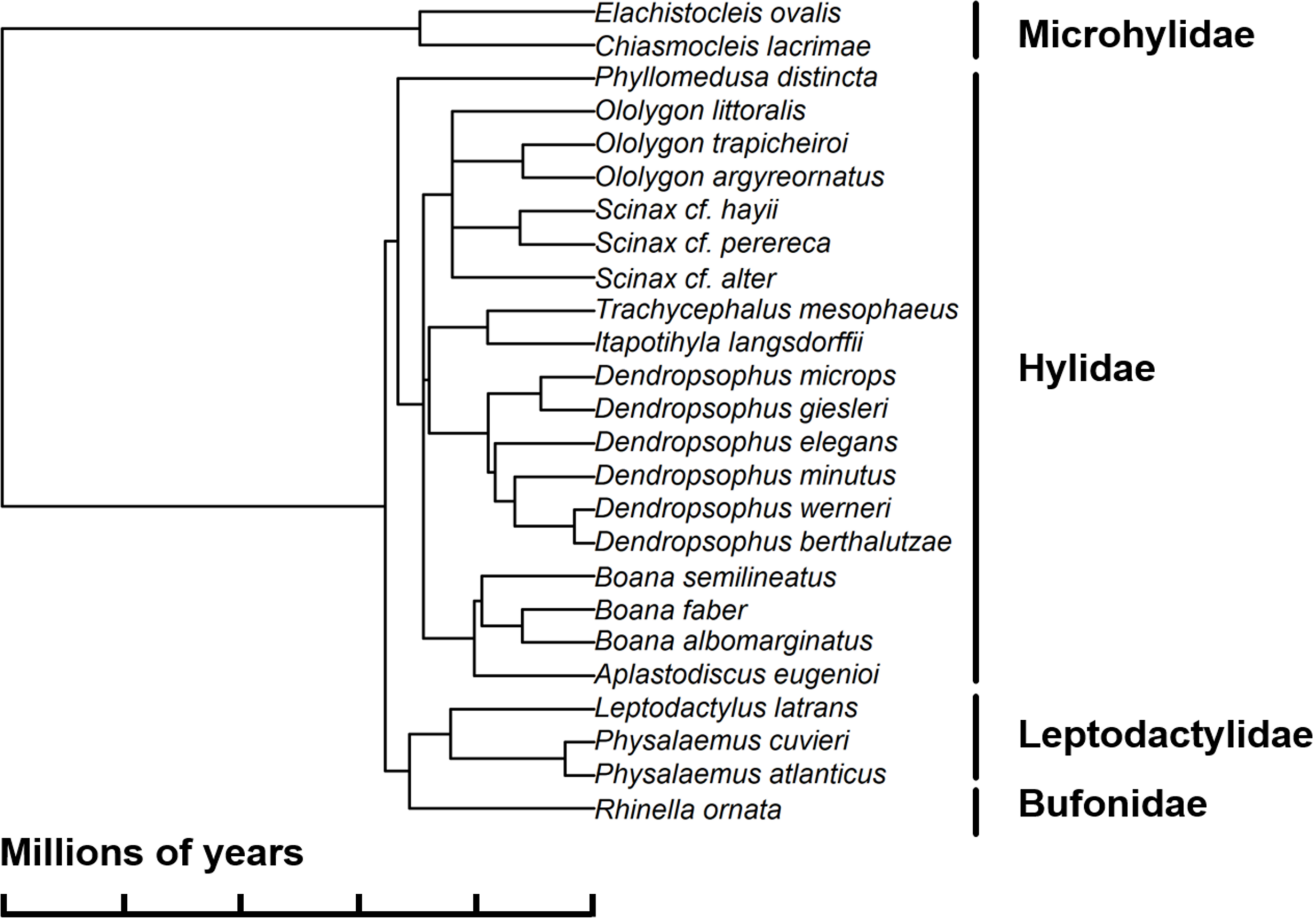
Phylogenetic relationships of anuran species recorded in the coastal plains of the state of São Paulo in Southeastern Brazil, based on a phylogenetic hypothesis proposed by [36]. Families are indicated on right. Time divergences were estimated from [36] and BLADJ algorithm (see text for details).

In order to estimate **PBD**, we constructed a pruned-tree based on the phylogenetic hypothesis of [36], which included only the species for our regional pool, which we considered to be all species recorded in the Serra do Mar coastal forests based on [37] (Fig 2). We assigned age estimates to all nodes based on [38]. Absent species were added to the original phylogeny of [36] by using the phylogenetic tools developed by [39]. We estimated tree branch lengths using the BLADJ algorithm [40], which was also used to evenly interpolate ages of dated to non–dated nodes.

In order to estimate **FBD,** we considered five ecomorphological attributes derived from nine morphological characters that are related to habitat use by tadpoles (see [28]). The ecomorphological attributes were: relative caudal height (RCH = (HCM + HDF + HVF)/BH); body compression (BC = BH/BTL), relative width of caudal musculature (RWCM = HCM/ MCW), relative caudal length (RCL = (BTL–BL)/BL), and relative spiracle size (RSP = SH/BH). Other ecomorphological attributes included were the following categorical measures: position of oral opening (OR), number of denticle rows (NDR), presence/absence of flagellum (FP), spiracle position (SP), eye position (EP) and body shape (BS) (for more details, see S1 Fig and S4 Table). Traits were selected that had strong associations with ecological and biological features, such as habitat use and foraging behaviour, which influence ecosystem structure and specific defence against predation [32, 41,42]. All attributes were used to construct a pairwise distance matrix of species. As we had binary (e.g. presence/absence of flagellum), categorical (e.g. eye position) and continuous (e.g. body compression) traits, we used the well-established Gower standardization for mixed variables [43].

### Processing spatial data

**Fig 3:**
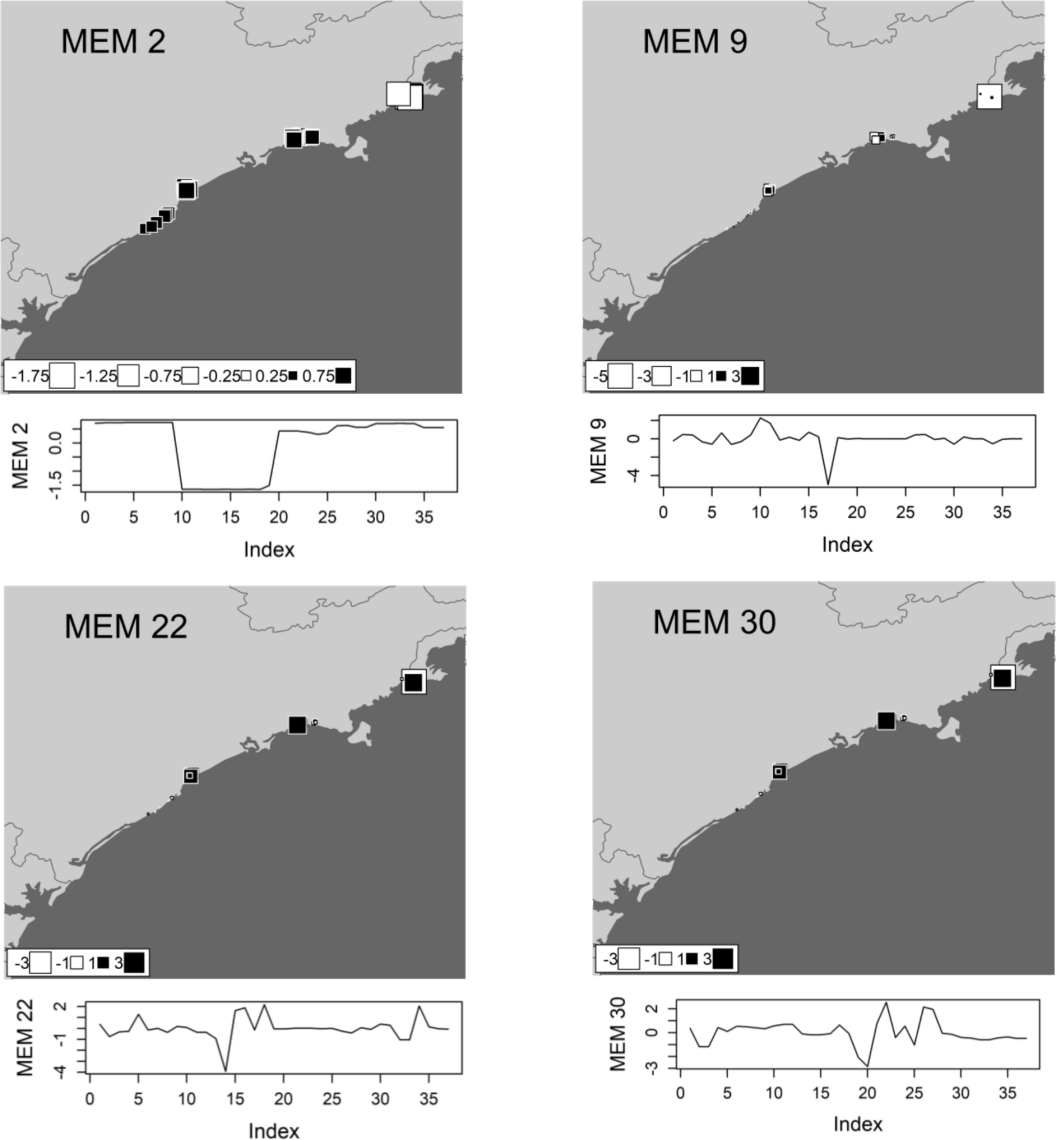
The moran eigenvector maps (MEMs) selected as the best model of spatial structure for functional beta diversity (FBD) of anuran species recorded in the coastal plains of the state of São Paulo in Southeastern Brazil. Each square represents a single pond sampled in the study region. White squares denote negative scores whereas black squares denote positive scores; the size of each square corresponds to the magnitude of its value. The values of these scores are also represented in a graph below each map, where it is possible to identify similarity in the periodicity among MEMs. MEM 2 represents positive autocorrelation in broad scales while MEMs 9, 22 and 30 represents negative autocorrelation in fine spatial structures of **FBD**.

**Fig 4:**
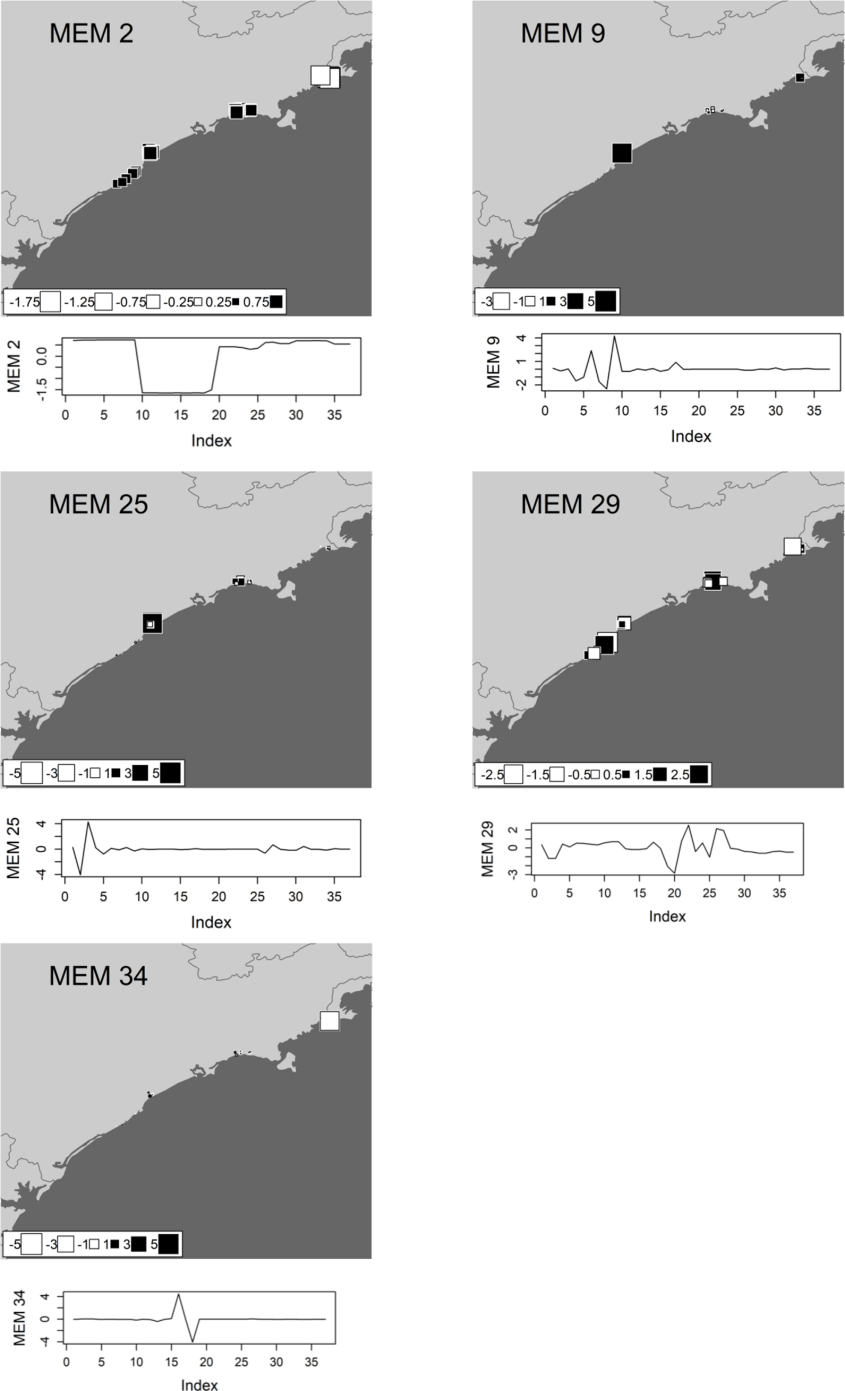
The moran eigenvector maps (MEMs) selected as the best model of spatial structure for taxonomic beta diversity (TBD) of anuran species recorded in the coastal plains of the state of São Paulo in Southeastern Brazil. **Each square represents a single pond sampled in the study region.** White squares denote negative scores whereas black squares denote positive scores; the size of each square corresponds to the magnitude of its value. The values of these scores are also represented in a graph below each map, where it is possible to identify similarity in the periodicity among MEMs. MEM 2 represents positive autocorrelation in broad scales while MEMs 9, 25, 29 and 34 represents negative autocorrelation in fine spatial structures of **TBD**.

**Fig 5:**
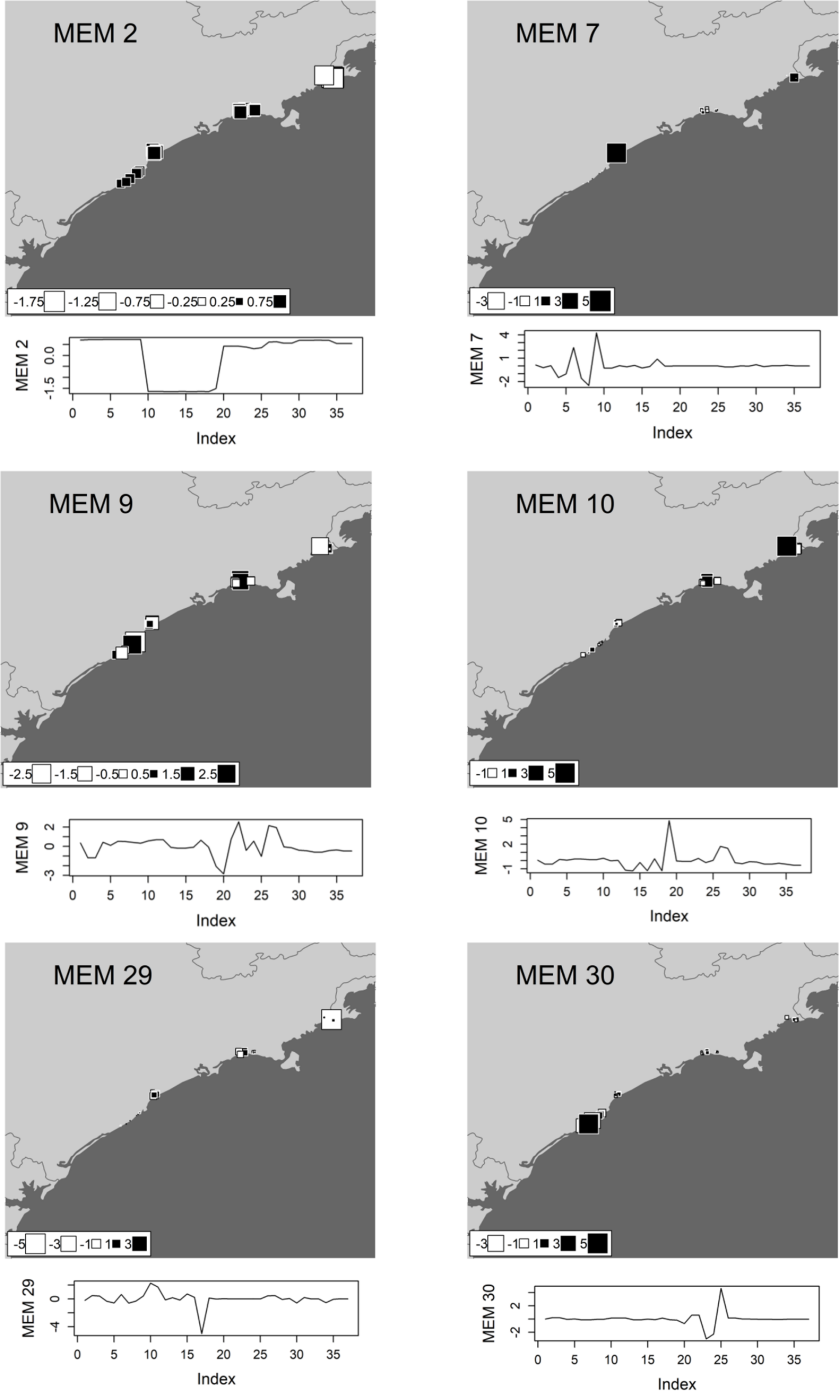
The moran eigenvector maps (MEMs) selected as the best model of spatial structure for phylogenetic beta diversity (PBD) of anuran species recorded in the coastal plains of the state of São Paulo in Southeastern Brazil. Each square represents a single pond sampled in the study region. White squares denote negative scores whereas black squares denote positive scores; the size of each square corresponds to the magnitude of its value. The values of these scores are also represented in a graph below each map, where it is possible to identify similarity in the periodicity among MEMs. MEM 2 represents positive autocorrelation in broad scales while MEMs 7, 9, 10, 29 and 30 represents negative autocorrelation in fine spatial structures of **PBD**.

We performed a spatial eigenfunction analysis to obtain spatial predictors and to describe the spatial structure of tadpole beta diversity (see [43,44]), based on Moran’s Eigenvector Maps (MEMs) [45]. MEMs describe multiscale spatial structures, ranging from fine to broad spatial scales, and determine which scales are more important in describing the spatial structure of response variables, which in our case were functional (**FBD**), taxonomic (**TBD**) and phylogenetic (**PBD**) diversities. This would then allow us to define submodels that represent different spatial scales and their associated MEMs (see [45]). We visually inspected each MEM that significantly defined a submodel of spatial structure of our study region. Two submodels were defined representing broad and fine spatial structures based on spatial patterns of selected MEMs eigenvectors, and the similarity in periodicity of spatial structure of significant MEMs (see the Moran’s Eigenvector Maps selected in Figs 3, 4 and 5). These two scales would represent spatially structured ecological and biogeographic processes, respectively, such as environmental control and dispersal.

The first step in using MEMs is the definition of a neighbourhood matrix, which describes the spatial relationships among objects [45]. In our case this meant defining which samples (ponds) are neighbours and which are not. We used a heuristic approach to assess several neighbourhood distances among ponds, starting with the minimum distance that spatially connects all ponds obtained through a minimum tree spanning algorithm (in our case, MST = 110.98 km) up to the maximum distance between samples (287.52 km). The MST can be defined as a neighbourhood matrix that connects all samples with the smallest total weight among various neighbourhood matrix. The three best neighbourhood matrices corresponded to each biodiversity component, selected from among fifty neighbourhood matrices tested, was as follows: for **FBD** = 273.11 km, **TBD** = 229.88 km and **PBD** = 161.42 km.

We assume that there is more ecological similarity between ponds spatially closer. However, our sampling effort was irregularly distributed along the studied region (Fig 1). Theses information must be take into account when we defined the spatial weights. We then constructed a spatial weighting matrix based on decreasing functions varying with distance, (i.e. **f**_1_ = 1 –*d*_*ij*_/max(*d*_*ij*_), in which *d*_*ij*_ denotes distance matrix between the *n* sampling locations). The best neighbourhood matrix was selected based on AICc. The better explanatory MEMs model for each beta diversity component were selected through stepwise model selection based on R^2^adj (Figs 3, 4 and 5).

### Measuring and partitioning functional, taxonomic and phylogenetic beta diversity

Based on Baselga [46] framework, we calculated pairwise Sørensen dissimilarity index of species (β_sor_), and decomposed into additive components account for nestedness component, represented by the Nestedness-resultant dissimilarity index (β_sne_) and spatial turnover component, represented by the Simpson pairwise dissimilarity index (β_sim_),. The β_sor_ accounting for total dissimilarity between samples (a monotonic transformation of beta diversity). β_sim_ represent for the pure identity replacement without the influence of richness difference. Finally, β_sne_ reflects the dissimilarity due to the difference in richness between assemblages. These indices are formulated as:

$$\beta_{sor}=\frac{b+c}{2a+b+c}$$

$$\beta_{sim}=\frac{min(b,c)}{a+min(b,c)}$$

$$\beta_{sne}=\frac{\max\left(b;c\right)-min(b,c)}{2a+b+c}\times \frac{a}{a+min(b,c)}$$

where *a* is the number of species common to both assemblages, *b* is the number of species in the first sample but not in the second while *c* is the number of species occurring in the second site but not in the first.

Additionally, based on Leprieur et al. [47] we computed the PhyloSor_sim_ and based on Villéger et al. [48] we calculated the FuncSor_sim,_ indices that represent the phylogenetic and functional component of pure spatial turnover between assemblages, respectively. These two dissimilarity indices are expressed as:

$${PhyloSor}_{sim}=\frac{min({PD}_{Tot}-{PD}_{k},{PD}_{Tot-{PD}_{j}})}{{PD}_{k}+{PD}_{j}-{PD}_{Tot}+min({PD}_{Tot}-{PD}_{k},{PD}_{Tot}-{PD}_{j}}$$

$${FuncSor}_{sim}=\frac{2\times \min\left(V(k),V(j))-2\times V(k\cap j\right)}{2\times \min\left(V(k),V(j))-V(k\cap j\right)}$$

where *PD* represents the Phylogenetic Diversity or the total branch length of a phylogenetic tree that contains all species present in an assemblage, *k* and *j* represent any two assemblages and *V* is the volume of the convex hulls in a multidimensional functional space. Therefore, in this paper, we analyse only the functional, taxonomical and phylogentic spatial tournover, namely FuncSor_sim_, β_sim_ and PhyloSor_sim_, respectively. These component represent the pure spatial variation of identity between assemblages, and hereafter they are referred as or functional distance matrix (**FBD**), taxonomic distance matrix (**TBD**), and phylogenetic distance matrix (**PBD**), respectively. Additionality, In order to understand the relationship among beta diversity components, we performed a Mantel test to assess the correlation between them [43].

### Statistical analyses

We performed a distance-based Redundancy Analysis (db-RDA) to analyse the explained variation of tadpole beta diversities (**FBD**, **PBD** and **PBD**) by pure environmental predictors, pure broad and fine-scale spatial predictors, their intersections and the residual variation expressed through the adjusted R^2^ statistics (R^2^adj) [35, 47]. Only variables selected through R^2^adj were included in variation partitioning, as explained above. We then used the variation partitioning approach to assess the shared and unique contributions of spatial and environmental predictors, and to determine which better explained variation in tadpole beta diversities [49]. Additionally, the significance of independent fractions was evaluated through permutation tests for distance-based Redundancy Analysis (db-RDA) [43].

## Results

The phylogenetic, taxonomical and functional beta diversity were significantly correlated with each other (S2 Fig!; P<0.05). Both environmental and spatial predictors (broad and fine-scales) were significantly related to all three components of beta diversity (P < 0,05). Using corrected AICc, we selected the best neighbourhood matrix and, through R^2^adj, we carefully chose the best spatial predictors for each component of diversity (Table 1). Based on the neighbourhood matrix, we calculated the spatial structure of beta diversity for 37 MEMs related to each component of biodiversity evaluated. One broad-scale MEM and two fine-scale MEMs were selected for functional beta diversity (**FBD)** (Fig 3), four fine-scale MEMs were selected for taxonomic beta diversity (**TBD)** (Fig 4), and one broad-scale MEMs and five fine-scale MEMs were selected for phylogenetic beta diversity (**PBD)** (Fig 5). The environmental models for **FBD**, **TBD** and **PBD** selected through stepwise model selection based on R^2^adj included pH, water conductivity, diversity of internal and external vegetation structure, canopy cover, presence of potential predator (fish) and water temperature (see S1A Table for further details).

**Table 1:** Best explanatory environmental and spatial models for taxonomic, phylogenetic and functional components of tadpole beta-diversity, selected by stepwise model selection based on R^2^adj (adjusted R squared) for environmental variables and spatial variables.

Beta diversity component	Environmental variables	R^2^adj	Spatial variables	R^2^adj
**Functional beta diversity (FBD)**	pH, diversity of internal vegetation structure, presence of potential predator (fish) and canopy cover, water conductivity,	0.35	MEM 2 (positive and from broad scale), and MEM 9 and 30 (negative and from fine scale)	0.32
**Taxonomic beta diversity (TBD)**	pH, water conductivity, diversity of internal vegetation structure, presence of potential predator (fish) and canopy cover	0.38	MEM 2 (positive and from broad scale), MEM 9, 25, 29 and 34 (negative and from fine scale)	0.41
**Phylogenetic beta diversity (PBD)**	diversity of external vegetation structure, canopy cover, water temperature and presence of potential predator (fish)	0.23	MEM 2 (positive and from broad scale), and MEM 7, 9, 10, 29 and 30 (negative and from fine scale)	0.39

**Fig 6:**
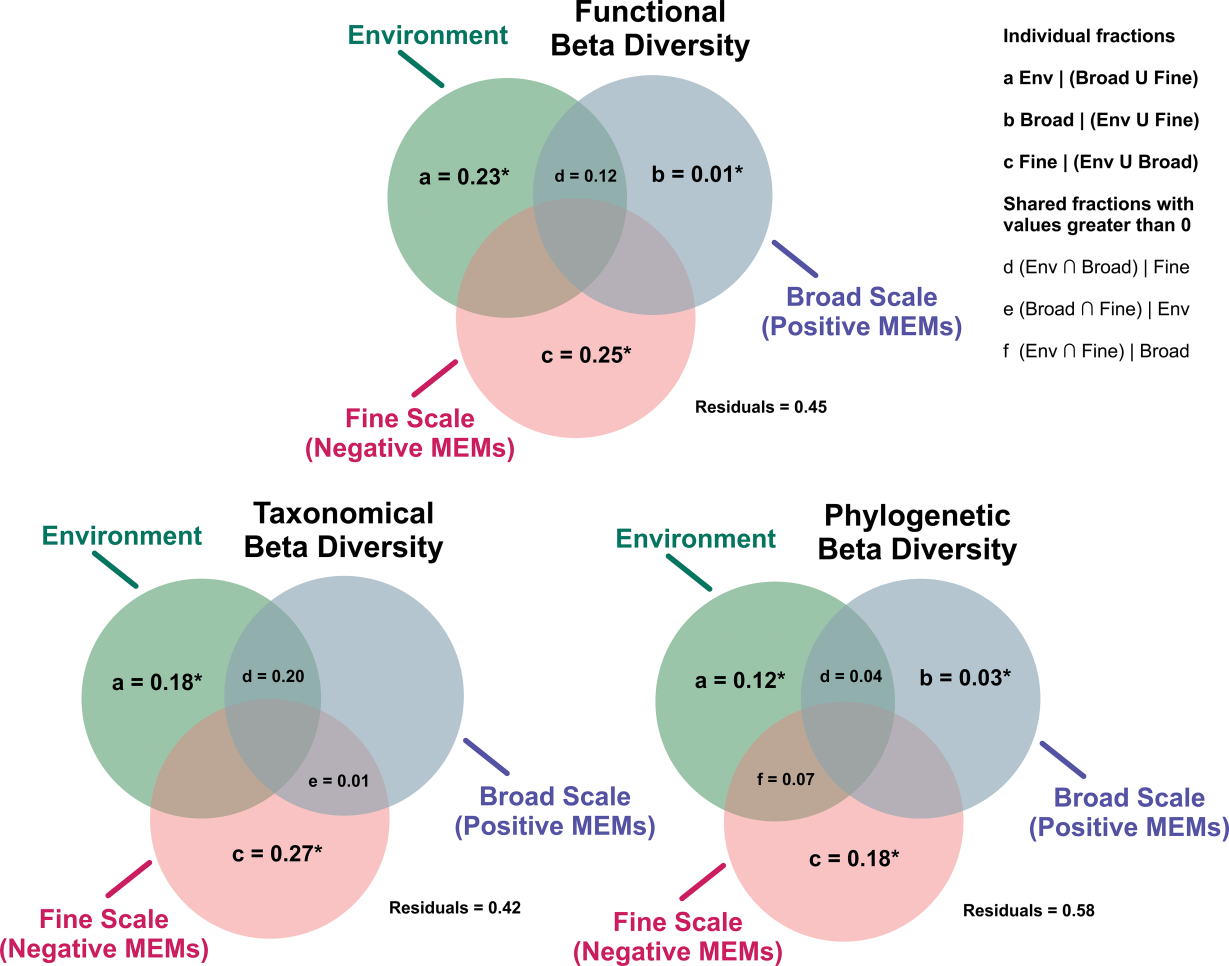
Partitioning of variation in taxonomic, functional and phylogenetic beta diversity of anuran species recorded in the coastal plains of the state of São Paulo in Southeastern Brazil, resulting from distance-based partial redundancy analysis (db-RDA and R^2^adj). The explained variation in the components of tadpole beta diversity was partitioned into shared and pure fractions of environmental, broad- and fine-scale spatial predictors. Fractions “a”, “b” and “c” represent pure effects of environment, broad-scale and fine-Scale predictors, respectively. Fractions “d” to “f” represent intersections or joint effects of different predictors. Residuals are the fraction not explained by any predictor included in the model. The upright box represents the notations to each set (i.e., fraction). The symbol “∩” represents “intersection”, “∪” represents “union”, and “|” represents “after controlling for”. Fractions with values lower than 0 are not shown in the diagram. Asterisks (*) denote significant fractions according to permutations tests (P < 0.05).

Partitioning of variation showed that 55% of the variation of **FBD** was explained by the full model (Fig 6), of which 23% was explained by pure environmental predictors, 12% by environmental variables structured in broad scales, 1% by broad-scale pure spatial predictors and 25% by fine-scale pure spatial predictors (Fig 6). 58% of variation **of TBD** was explained by selected predictors, with 18% explained by pure environmental predictors, 27% explained by fine-scale pure spatial predictors and 20% by environmental variables structured in broad scales and 1% by the intersection between broad and fine-scales spatial predictors(Fig 6). Regarding the variation of **PBD,** 49% was explained by the full model, of which 12% was explained by pure environmental predictors, 17% by environmental variables structured in fine scales, 4% by environmental variables structured in broad scales, 3% by broad-scale pure spatial predictors, and18% by fine-scale spatial predictors (Fig 6).

There were relative different responses of tadpole communities to environmental and spatial predictors when spatial turnover was partitioned into functional, taxonomical and phylogenetic components. Functional beta diversity exhibited a more proportionally relationship with pure fractions of environmental and spatial predictors (**FBD:** environment = 23%, space = 25%, Fig 6). However, if considered the all fractions together (pure and spatially structured)habitat predictors explained more **FBD** and **TBD** than **PBD** (**FBD** = 35%, **TBD** = 38%, **PBD** = 23% Fig 6) On the other hand, broad-scale spatial predictors were, although weakly, associated with better with phylogenetic than functional beta diversity (**PBD: broad** = 3%, **FBD: broad =** 1%, Fig 6).

## Discussion

We found the three components of beta diversity studied here to be structured in a complex and sometimes distinct way by spatial and environmental predictors. The fine-scale negative MEMs were proportionally the most important predictors to **PBD TBD** and **FBD**. Moreover, taxonomic beta diversity (**TBD**) was similarly explained by fine-scale spatial predictors and fine-scale environmental predictors. Pure and spatially structured fraction of environmental predictors explained an important part of variation of all beta diversity components, although it was more proportionally important to **FBD** and **TBD** (for more details, Fig 6). Additionally, the three beta diversity component were found high correlated each other. This make sense since they are decomposed from the same community data and seems to be structure by the similar processes although with different relative importance.

Nevertheless, we found strong spatial structure in all three components of beta diversity assessed herein, as evidenced by the larger variation in components of tadpole beta diversities explained by pure spatial predictors. The more important and numerous spatial predictors for functional, taxonomic and phylogenetic beta diversities were at fine-scales, as illustrated by MEM 30 (**FBD**), MEMs 29 (**TBD)** and MEM 25 (**PBD**) (Figs 3, 4 and 5). These results suggest that a large portion of the variation in beta diversity is explained by processes structured at fine-scales (Table 1, Figs 3, 4 and 5). Furthermore, the fine scales MEMs selected has negative eigenvalues, representing negative autocorrelation (white squares, Figs 3,4 and 5) [49]. However, for functional and phylogenetic component, broad-scale spatial predictors with positive eigenvalues were also selected (MEM 2 (**FBD**) and MEM 2 (**PBD**), Figs 3 and 6), indicating that there is still spatial structure in more regional scales related to positive spatial correlation. It worth to note that, although the variance partition did not select significant R^2^ to the **TBD** broad scale fraction (Fig 6), the same broad scale spatial predictor (MEM 2) was selected for the three components. This spatial predictor is virtually splitting the northern region samples from the others (Figs 3, 4 and 5), which make sense since the last is supposed more diverse in species composition (for details, see [9]), showing that there is an important ecological difference between the northern assemblages when compared to the others.

These findings suggest that two distinct spatially structured processes might be structuring the three components of beta diversity studied. The first of these could be the differences in spatially structured environmental variables among samples [43], which were not directly measured in this study but possibly affect anurans, and include variables such as hydroperiod or distance from the nearest vegetal formation [50]. Since these variables are probably spatially structured, their effects, and relative explanation of variation in beta diversity, may be represented by pure fine scale spatial components [26,51].

On the other hand, pure spatial effects could indicate the influence of neutral processes related to random and limited dispersal of species. Neutral theory postulates that similarity among sites decreases with increasing geographic distance, thereby generating spatial structure in beta diversity related to random dispersal [7,26]. Anurans are generally considered to have low dispersal abilities [52], which may in fact prevent species from reaching suitable habitats and, consequently, determine tadpole distributions [53,54]. For instance, spatial predictors explained 18.5% of anuran taxonomic beta diversity in Amazonian communities [26] and 21% in Lowlands Atlantic Forest [9]. Amazonian communities were consistent with neutral predictions based on comparisons of simulated communities structured exclusively by neutral dynamics [26]. However, a study of the Atlantic Forest frogs beta diversity [9], showed that pure spatial fractions are not consistent with what would be expected by neutral dynamics. Therefore, the spatial fractions of our study cannot be interpreted unambiguously, as they may reflect the differences of spatially-structured, but non-measured, environmental variables among samples, biotic interactions or random dispersal processes, generating negative spatial autocorrelation in ecological communities. However, the above cited paper of frogs beta diversity on the same study region [9] showed that the spatial fractions of explained variation are not congruent with what would be expected by neutral dynamics, indicating that spatial predictors may be representing the effect of unmeasured environmental variables on communities.

Despite significant portions of variation in the components of anuran beta diversity being explained by spatial and environmental predictors, great portions remained unexplained (**FBD** = 45%, **TBD** = 42% and **PBD** = 58%; Fig 6). Anuran beta diversity in the study region may also be driven by stochastic mechanisms, which supposes that population dynamics is not dependent on environmental parameters but regulated by ecological drift and/or random dispersal [7]. In addition, non-measured pure environmental or biological predictors may also be related to residual variation. Competition, for instance, is historically recognized as an important factor in structuring diversity and abundance at local scales (e.g., [51]). Furthermore, non-measured climatic variables structured on broad-scales, such as temperature and humidity levels, are important to the biology and ecology of adult anurans [23, 55], and may be part of the unexplained portion of the variation in our beta diversities.

Although spatial predictors were found to be more important in describing beta diversity in our study, habitat variables (pure environmental fraction and environmental variables spatially structured in fine scales) were also significantly related to variation in beta diversity (**TBD** = 38%, **FBD** = 35% and **PBD** = 23%, Fig 6). Anurans are particularity affected both directly and indirectly by environmental conditions at different spatial scales (see [22, 56, 57]). For instance, vegetation structure, hydroperiod (at local scales) and temperature and humidity levels (at regional scales), influence several aspects of amphibian ecology, including species richness and composition, and even functional and phylogenetic diversity [56–58]. Along the Atlantic Forest coastal plains, climatic variables seem to be particularly influential on anuran beta diversity, although less importantly than the spatial structure of diversity patterns [9]. It is worth noting that the relationships of **FBD** with both pure environmental and fine-scale spatial predictors were similarly important (Fig 6), indicating that both ecological and spatial processes at local-scales should also affect functional beta diversity. Furthermore, environmental predictors are commonly invoked to infer niche–based processes, given the strong relationship between species distributions and environmental variables [59].

The most important environmental variables selected that was related to phylogenetic, taxonomic and functional components of beta diversity was the vegetation structure of ponds, presence of potential fish predators, and canopy cover (Table 1). As stated by Niche Theory, functional traits are crucial to interactions between species and the environment [6], indicating that the diversity of the vegetation structure of ponds is a key environment variable interacting with species traits and consequently determining their occurrence and generating a response in all components of beta diversity. This variable was also an important predictor of phylogenetic structure of anurans in the study region [60], and is particularly relevant to species occurrence in ponds. The diversity of vegetation structure may affect anuran assemblages by providing sites for vocalization and/or oviposition for adults, or protection from predators for tadpoles [20,57,61]. In the coastal plains, we found several hylid species in the ponds sampled, such as *Scinax* spp. and *Dendropsophus* spp., which are associated to greater diversity of vegetation structure, and so indicate the importance of this variable as a reproductive resource for those species [33,57].

Canopy cover is known to have an indirect influence locally by virtually splitting species associated with forests, such as microhylids, from those associated with open areas, such as some hylids including *Dendropsophus* spp. and *Boana* spp. [33,62] present study. This can be explained by the fact that the higher productivity in open canopy ponds provides greater resource availability, and in turn could influence anuran diversity and species performance [62–64]. In the coastal plains studied here, we found that canopy cover is related to **FBD**, **TBD** and **PBD** (Table 1). We also found that it may affect phylogenetic and functional structure of tadpoles in the study region [60] and thus it seems to be a key environmental variable affecting not just the structure of anuran communities in coastal plains. Additionally, the presence of potential fish predators was also especially related to **PBD, TBD** and **FBD.** This is not surprising, since fish predators are known to negatively affect development and growth, as well as induce morphological modifications in tadpoles [65–67]. However, the particular relationship of the presence of fish predators with **PBD** of tadpoles needs to be further investigated. Nevertheless, in our study region we observed the absence of certain species or clades, such as *Elaschistocleis ovalis*, *Chiasmocleis carvalhoi*, and *Dendropsophus* spp., to be associated with the presence of fish.

Although not equally important for all components of beta diversity, other local environmental variables were also selected as predictors of **FBD, TBD** and **PBD**. (Table 1). For instance, pH was selected as important for FBD and TBD. In fact, it can directly influence development, fitness, survivor and even the occurrence of tadpoles in different assemblages [25,28]. Indeed, we observed different species of *Scinax*, *Physalaemus*, Leptodactylidae and Microhylidae to be associated with ponds with a more neutral pH (7.0), and not with those with lower pHs (< 5.0), virtually separating some species from others. Water conductivity is assumed to be a surrogate for food availability and productivity in aquatic communities [68]. Productivity directly influences local species diversity because increasing productivity usually generates greater complementarity in resource use, and consequently the higher co-occurrence of species (e.g., [63]). We found that certain species, such as *Dendropsophus* spp. and *Rhinella* spp., were associated with higher conductivity, and that leptodactilids were associated more with lower conductivity. Such a relationship seems to be also significant for the taxonomic structure of anuran tadpoles [60] indicating that conductivity is indirectly linked to the occurrence of specific species in the coastal plains and then it is an important variable in assessing the structure of anuran communities.

We found that water temperature is important for **PBD**, which could raise some possible explanations about this relationship. For instance, temperature and humidity levels influence several aspects of amphibian ecology, including species richness and composition, and even phylogenetic diversity [23,55], then, water temperature, higher related with air temperature, could also be influencing the phylogenetic diversity. However, further investigations are necessary to test those assumptions.

Although all components of beta diversity showed a strong spatial structure, **PBD** and **TBD** seem to have a broad spatially structure of beta diversity, although much less important. (Fig 6). This makes sense since **PBD** represent lineages turnover and can be related more to evolutionary and historical processes that generally are strongly spatial structured, such as allopatric speciation and dispersal [4]. Furthermore, potential differences of local unmeasured environmental predictors may be controlling the lineages representativeness in samples [43], generating the negative autocorrelation found here at fine scales.

Although an important fraction of the best environmental predictors for **PBD** and **FBD** were spatially structured, habitat variables was proportionally important to all biodiversity components (Fig 6). This also makes sense since all these components of diversity are related to ecological factors that can determine the occurrence of species in local assemblages, such as environmental control [11, 69]. Spatial processes at fine-scales, representing negative autocorrelation, were specially significantly related to all beta diversity components assessed herein, indicating that the major portion of beta diversity is structured among local communities. In fact, fine-scale habitat heterogeneity was observed acting as ecological filters on two Amazonian species, *Allobates sumtuosus* and *Atelopus spumarius* [25], indicating the potential effect of environment variables structured at local scales driving species occurrence in assemblages.

We should caution that spatial and environmental structure may be reflected differently in each component of diversity, and therefore all possible facets of biodiversity need to be analysed in order to assess patterns of diversity of a given region and to explore possible underlying processes. For instance, it is accepted that functional diversity is directly affected by assembling processes, as it essentially represents the interaction between the traits of an organism and the environment [11,12]. For the last few decades, phylogenetic diversity has been used as a proxy for functional diversity in the assessment of the assembling processes of communities, under the assumption of phylogenetic niche conservatism. However, in the study region, we found a complex set of drivers of beta diversity, as different processes seem to regulate **FBD, TBD** and **PBD** (e.g. relatively distinct amount of environment explanation for **FBD** and **PBD**, Fig 6), indicating that, phylogenetic diversity is not necessarily a suitable proxy for functional diversity, as advocated by some authors [70,71]. We then believe that our results provide empirical support to the assumption that functional diversity could be a better indicator of local community assembly processes than phylogenetic diversity [27,72,73]. In this sense, we highlight the importance of actually testing the assumption of phylogenetic niche conservatism in order to avoid erroneous conclusions.

We are aware that our approach has limitations, since correlation does not necessarily represent cause and effect relationships, and care must be taken in the interpretation of the explanatory fractions of each beta diversity component [72,73]. Nonetheless, our study is an important step toward revealing the processes driving the distinct components of beta diversity in vertebrate communities, and the structure of different facets of biodiversity in the Atlantic Forest. The significant spatial structure in the components of anuran beta diversity found in our study was consistent, although in different magnitudes, with other tadpole communities and also other aquatic organisms, including fish, macroinvertebrates and zooplankton, among others [17, 50,74–78].

Although we could not establish dispersal-based processes as a determining factor of beta diversity, the spatial pattern found in these aquatic communities seems to reflect a noteworthy spatial structure on a continuum as a result of fine to broad-scale processes. Finally, we highlight the contrast we found in the assessment of different components of biodiversity, with spatial predictors at fine-scales (representing negative autocorrelation) showing great influence on all beta diversity components, but specially for **FBD** and **TBD**. On the other hand, local environmental predictors seem to be also important, explaining a significant variation in all beta diversity components, but particularly to **FBD** and **TBD**. These outcomes seem to reflect the complex way that spatial and niched-based processes should influence historically and ecologically each component of biodiversity.

## Supporting information

S1 Fig Morphological characters description. (DOCX)

S2 Fig Mantel correlations between the functional, taxonomic, phylogenetic spatial turnover. (DOCX)

S1 Table Environmental variables description. (DOCX)

S2 Table Anura species occurrence recorded in the coastal plains. (DOCX)

S3 Table Raw spatial and environmental data recorded from sites in the coastal plain. (DOCX)

S4 Table Raw ecomorphological data from anura species recorded in the coastal plain. (DOCX)
