# Supplementary material for: The complex roles of space and environment in structuring functional, taxonomic and phylogenetic beta diversity of frogs in the Atlantic Forest
Source: PLoS One. 2018 Apr 19;13(4):e0196066. doi: 10.1371/journal.pone.0196066 (PMC5908149; doi:10.1371/journal.pone.0196066)

**S1 Fig. Morphological characters description.** The attributes was recorded through calculation of ecomorphological attributes: body width (BW), musculature caudal width (MCW), height of caudal musculature (HCM), height of dorsal fin (HDF), height of ventral fin (HVF), body length (BL), body height (BH), spiracle’s height (SH), and body total length (BTL).


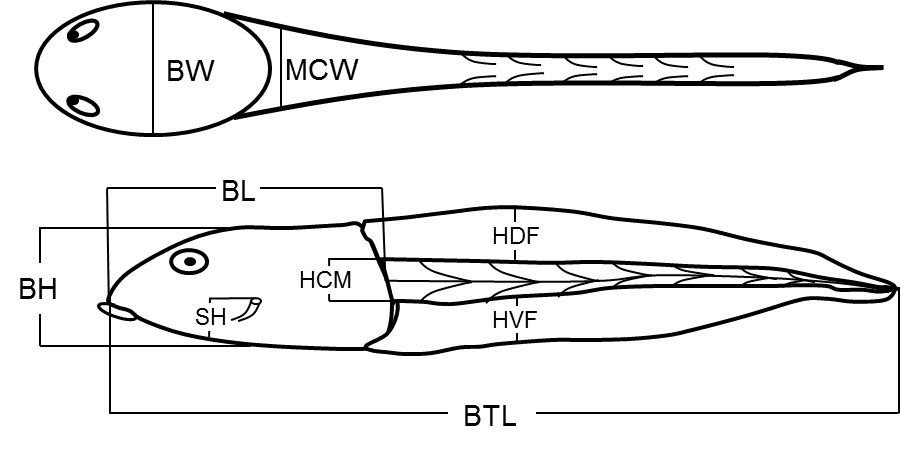

Supplement: S1 Fig — (DOCX) [file pone.0196066.s001.docx]
