# Supplementary material for: The complex roles of space and environment in structuring functional, taxonomic and phylogenetic beta diversity of frogs in the Atlantic Forest
Source: PLoS One. 2018 Apr 19;13(4):e0196066. doi: 10.1371/journal.pone.0196066 (PMC5908149; doi:10.1371/journal.pone.0196066)

**S2 Fig. Mantel correlations (r) between the functional, taxonomic, phylogenetic spatial turnover.** All correlation was calculated over pairs of samples. Significant correlations are indicated by italic text (999 permutations).


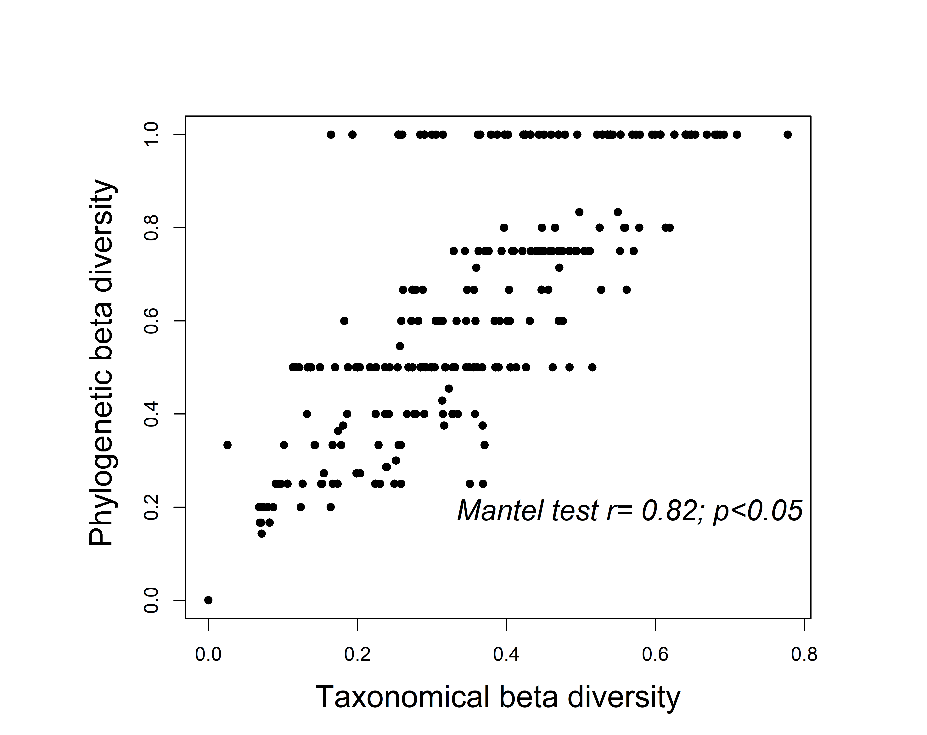

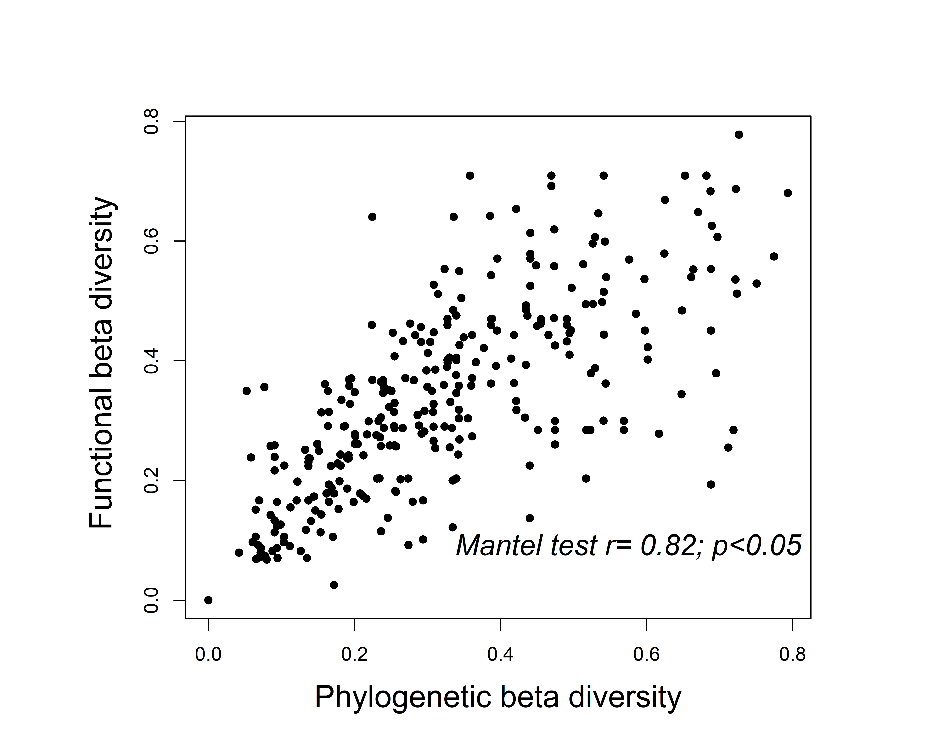

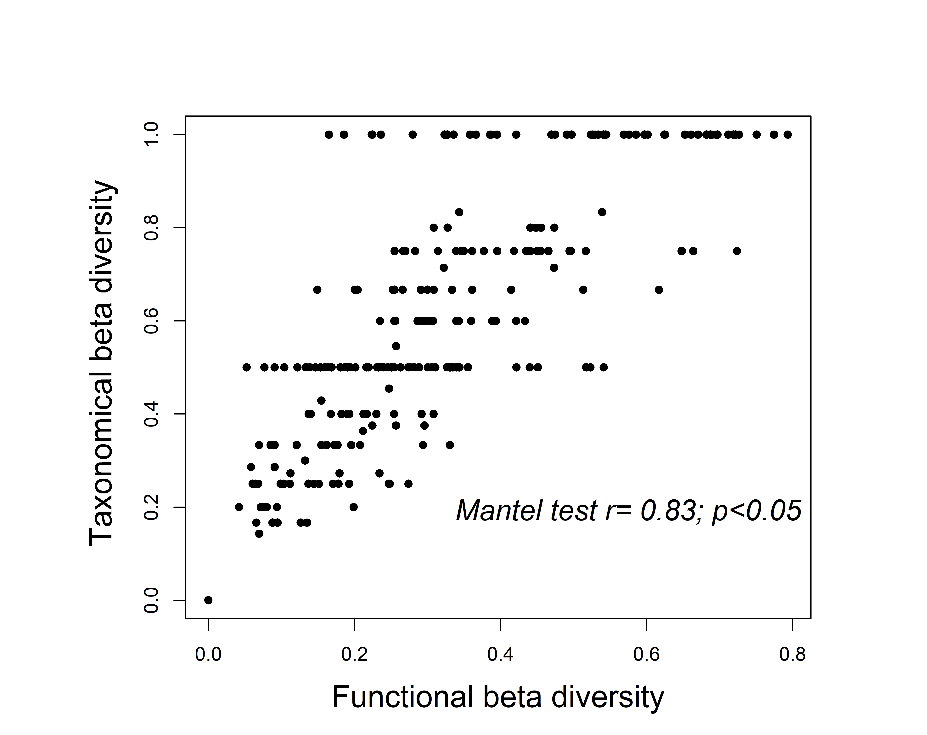

Supplement: S2 Fig — (DOCX) [file pone.0196066.s002.docx]
