# Supplementary material for: The complex roles of space and environment in structuring functional, taxonomic and phylogenetic beta diversity of frogs in the Atlantic Forest
Source: PLoS One. 2018 Apr 19;13(4):e0196066. doi: 10.1371/journal.pone.0196066 (PMC5908149; doi:10.1371/journal.pone.0196066)
Supplement: S1 Table — (DOCX) [file pone.0196066.s003.docx]

**S1 Table. Environmental variables description**. The variables was recorded in 37 ponds sampled in the coastal plains in Southeastern Brazil.

| **Variable** | **Parameter description** |
| --- | --- |
| **Hydroperiod** | *ephemeral*: retained water for a period of time less than sample period; *temporary*: retained water throughout the entire sampling period, but not for 100% of the year; *permanent*: retained water throughout the entire sampling period and throughout the year |
| **Area** | surface area of the pond (m^2^) |
| **Water depth** | maximum depth of the pond (cm) |
| **Canopy cover** | percentage of canopy cover above the pond |
| **Diversity of internal vegetation structure** | percentage of each vegetation type present in the pond (macrophytes submerged, floating and upright herbaceous, shrubby and arboreal vegetation) |
| **Diversity of external vegetation structure** | percentage of each vegetation types present around the pond (upright herbaceous, shrubby and arboreal vegetation) |
| **Presence of potential predator (fish)** | collected by a dipnet: absent (0) or present (1) |
| **pH** | pH of the pond water |
| **Dissolved oxygen** | dissolved oxygen of the pond water (ppm) |
| **Water conductivity** | Level of conductivity of the pond water (µs/cm) |
| **Water temperature** | temperature of the pond water (°C) |
