# Supplementary material for: The complex roles of space and environment in structuring functional, taxonomic and phylogenetic beta diversity of frogs in the Atlantic Forest
Source: PLoS One. 2018 Apr 19;13(4):e0196066. doi: 10.1371/journal.pone.0196066 (PMC5908149; doi:10.1371/journal.pone.0196066)
Supplement: S3 Table — (DOCX) [file pone.0196066.s005.docx]

**S3 Table. Raw spatial and environmental data recorded from sites in coastal plain.** WD = water depth; int. div. = diversity of internal vegetation structure - 0 (no vegetation), 1 (maximum vegetation complexity); ext. div. = diversity of external vegetation structure - 0 (no vegetation), 1 (maximum vegetation complexity); fish= 0 (absence), 1 (presence); DO = dissolved oxygen; WC = water conductivity; WT= water temperature (for more details, see S1 Table).

| Sites | longitude | latitude | area (m) | WD (m) | canopy cover (%) | int. div. | ext. div. | fish | pH | DO (%) | WC (µS/cm) | WT (°C) |
| --- | --- | --- | --- | --- | --- | --- | --- | --- | --- | --- | --- | --- |
| site1 | -46.9394 | -24.1986 | 532.98 | 0.27 | 0.00 | 0.44 | 0.84 | 0 | 6.37 | 9.63 | 103.27 | 31.33 |
| site2 | -46.8946 | -24.2173 | 583.73 | 0.55 | 61.61 | 0.62 | 0.63 | 0 | 4.34 | 9.60 | 116.80 | 27.83 |
| site3 | -46.8944 | -24.2249 | 12968.2 | 1.50 | 0.00 | 0.21 | 0.64 | 1 | 5.51 | 8.77 | 72.47 | 32.03 |
| site4 | -46.9264 | -24.2341 | 235.5 | 0.45 | 80.96 | 0.24 | 0.43 | 0 | 4.45 | 9.93 | 117.60 | 28.37 |
| site5 | -46.9144 | -24.2407 | 1200.11 | 0.25 | 0.00 | 0.81 | 0.43 | 0 | 4.80 | 8.33 | 109.84 | 32.92 |
| site6 | -46.9314 | -24.2413 | 213.77 | 0.55 | 51.48 | 0.22 | 0.43 | 1 | 7.01 | 11.03 | 102.83 | 30.00 |
| site7 | -46.931 | -24.2463 | 9796.8 | 2.00 | 0.00 | 0.01 | 0.43 | 1 | 5.83 | 11.73 | 108.37 | 32.93 |
| site8 | -46.9265 | -24.2326 | 368.2 | 0.02 | 69.81 | 0.62 | 0.44 | 1 | 4.61 | 10.10 | 95.37 | 32.13 |
| site9 | -46.9221 | -24.235 | 251.56 | 0.05 | 63.51 | 0.62 | 0.43 | 0 | 6.47 | 4.37 | 59.10 | 32.10 |
| site10 | -44.8533 | -23.3523 | 7850 | 0.50 | 40.34 | 0.85 | 1.00 | 0 | 6.48 | 8.97 | 60.80 | 27.80 |
| site11 | -44.8498 | -23.3602 | 3656.84 | 1.30 | 57.98 | 0.05 | 0.83 | 1 | 7.67 | 6.63 | 74.00 | 28.83 |
| site12 | -44.8507 | -23.3589 | 288.82 | 0.60 | 69.33 | 0.45 | 0.24 | 0 | 6.49 | 9.70 | 68.60 | 28.23 |
| site13 | -44.8327 | -23.3593 | 183.69 | 0.10 | 0.00 | 0.84 | 1.00 | 0 | 6.50 | 5.70 | 27.60 | 35.07 |
| site14 | -44.8266 | -23.3563 | 838.38 | 0.60 | 72.21 | 0.81 | 1.00 | 0 | 6.85 | 10.60 | 77.33 | 25.23 |
| site15 | -44.8453 | -23.3448 | 9721.44 | 2.00 | 36.98 | 0.85 | 0.84 | 1 | 6.98 | 5.87 | 33.33 | 29.07 |
| site16 | -44.8324 | -23.3644 | 12560 | 0.50 | 0.00 | 1.00 | 0.84 | 0 | 6.50 | 4.23 | 41.47 | 31.53 |
| site17 | -44.8536 | -23.3564 | 2512 | 0.25 | 82.12 | 0.65 | 1.00 | 0 | 7.01 | 5.03 | 69.30 | 27.30 |
| site18 | -44.8326 | -23.3654 | 38.43 | 0.20 | 93.45 | 0.05 | 1.00 | 0 | 6.76 | 8.53 | 85.57 | 25.73 |
| site19 | -44.9400 | -23.3312 | 267214 | 2.00 | 0.00 | 0.25 | 1.00 | 1 | 7.24 | 3.50 | 76.87 | 30.53 |
| site20 | -47.0903 | -24.4444 | 6441.71 | 1.00 | 64.73 | 0.05 | 1.00 | 1 | 7.09 | 5.23 | 76.13 | 25.90 |
| site21 | -47.1104 | -24.4621 | 1417.87 | 0.80 | 74.15 | 0.83 | 0.64 | 1 | 7.09 | 8.43 | 236.33 | 24.37 |
| site22 | -47.1266 | -24.4747 | 2229.4 | 4.00 | 0.00 | 0.43 | 0.84 | 1 | 7.50 | 7.20 | 51.70 | 27.73 |
| site23 | -47.2039 | -24.5338 | 3579.6 | 0.95 | 0.00 | 0.44 | 1.00 | 1 | 6.33 | 12.10 | 53.50 | 27.60 |
| site24 | -47.3118 | -24.5927 | 498.87 | 1.50 | 68.87 | 0.65 | 1.00 | 0 | 7.33 | 9.97 | 119.23 | 27.03 |
| site25 | -47.2478 | -24.5728 | 3552.97 | 0.65 | 42.68 | 0.85 | 1.00 | 1 | 7.09 | 13.37 | 83.23 | 25.83 |
| site26 | -45.8699 | -23.7168 | 49455 | 2.00 | 0.00 | 0.45 | 0.84 | 1 | 6.62 | 5.86 | 79.56 | 26.53 |
| site27 | -45.8655 | -23.7433 | 28138.17 | 1.50 | 0.00 | 0.45 | 0.84 | 0 | 6.93 | 3.96 | 85.96 | 27.43 |
| site28 | -45.7674 | -23.731 | 2145.37 | 1.10 | 0.00 | 0.45 | 0.84 | 1 | 6.45 | 8.50 | 71.33 | 32.60 |
| site29 | -45.7634 | -23.7438 | 502.4 | 1.10 | 54.13 | 0.01 | 0.84 | 1 | 6.68 | 7.63 | 72.90 | 26.70 |
| site30 | -45.9322 | -23.7299 | 314 | 0.30 | 80.11 | 0.03 | 0.64 | 1 | 6.34 | 7.80 | 71.70 | 24.83 |
| site31 | -45.9312 | -23.7355 | 753.6 | 1.20 | 68.60 | 0.45 | 1.00 | 1 | 5.74 | 5.40 | 71.10 | 28.00 |
| site32 | -45.9265 | -23.7544 | 803.84 | 1.10 | 64.06 | 0.63 | 0.84 | 1 | 4.48 | 9.23 | 67.70 | 31.63 |
| site33 | -45.927 | -23.7544 | 282.6 | 0.80 | 0.00 | 0.64 | 1.00 | 1 | 4.68 | 3.20 | 64.57 | 28.97 |
| site34 | -45.9163 | -23.7620 | 1099 | 1.60 | 33.30 | 0.85 | 0.84 | 1 | 5.97 | 6.93 | 76.37 | 33.90 |
| site35 | -45.7528 | -23.7265 | 3140 | 0.40 | 0.00 | 0.65 | 0.84 | 0 | 6.12 | 5.90 | 58.10 | 24.10 |
| site36 | -45.7476 | -23.7336 | 1570 | 1.10 | 77.63 | 0.25 | 1.00 | 1 | 6.24 | 9.00 | 43.83 | 23.70 |
| site37 | -45.7471 | -23.7362 | 4710 | 0.60 | 0.00 | 0.00 | 1.00 | 0 | 5.73 | 2.80 | 51.80 | 24.03 |
