# Supplementary material for: The complex roles of space and environment in structuring functional, taxonomic and phylogenetic beta diversity of frogs in the Atlantic Forest
Source: PLoS One. 2018 Apr 19;13(4):e0196066. doi: 10.1371/journal.pone.0196066 (PMC5908149; doi:10.1371/journal.pone.0196066)
Supplement: S4 Table — (DOCX) [file pone.0196066.s006.docx]

**S4 Table. Raw Ecomorphological data from Anura species recorded in coastal plain.** Oral opening: 0 (ventral), 1 (anteroventral), 2 (anterior); Flagellum: 0 (absence), 1 (presence); Eye position: 0 (dorsal), 1 (dorsolateral), 2 (lateral); Body format 0 (globular), 1 (depressed/globular), 2 (elongate/globular), 3 (triangular), 4 (depressed /triangular) 5 (elongate/triangular); Denticules: number of denticules rows; HCM: height of caudal musculature; HDF: height of dorsal fin; HVF: height of ventral fin; BH: body height; BW: body width; BL: body length; MCW: musculature caudal width; SH: spiracle’s height; BLT: body total length.

| species | Oral opening | Flagellum | Eye position | Body format | Denticules | HCM | HDF | HVF | BH | BW | BL | MCW | SH | BLT |
| --- | --- | --- | --- | --- | --- | --- | --- | --- | --- | --- | --- | --- | --- | --- |
| *Elachistocleis ovalis* | 2 | 0 | 1 | 1 | 0 | 1.176 | 0.543 | 0.48 | 2.028 | 3.373 | 4.544 | 1.181 | NA | 13.944 |
| *Chiasmocleis carvalhoi* | 2 | 1 | 2 | 2 | 0 | 1.268 | 1.336 | 1.713 | 3.839 | 4.915 | *7.132* | 8.764 | NA | 15.896 |
| *Dendropsophus berthalutzae* | 2 | 1 | 2 | 2 | 0 | 1.471 | 1.061 | 1.023 | 2.713 | 3.234 | 5.341 | 1.316 | 1.378 | 15.9267 |
| *Dendropsophus elegans* | 2 | 1 | 2 | 4 | 0 | 1.509 | 1.031 | 1.183 | 2.630 | 2.783 | 4.699 | 1.257 | 1.270 | 14.3084 |
| *Dendropsophus giesleri* | 2 | 1 | 2 | 2 | 0 | 1.185 | 1.191 | 1.085 | 2.364 | 2.704 | 3.904 | 1.160 | 0.753 | 11.914 |
| *Dendropsophus microps* | 2 | 1 | 2 | 5 | 0 | 1.300 | 0.780 | 1.015 | 2.028 | 2.430 | 3.303 | 0.718 | 0.703 | 9.9864 |
| *Dendropsophus minutus* | 2 | 1 | 2 | 4 | 0 | 1.317 | 1.156 | 1.221 | 3.040 | 2.758 | 4.343 | 1.217 | 1.047 | 13.9034 |
| *Dendropsophus werneri* | 2 | 1 | 2 | 1 | 0 | 1.122 | 1.208 | 0.723 | 1.918 | 2.272 | 3.558 | 1.149 | 0.772 | 12.7596 |
| *Aplastodiscus eugenioi* | 2 | 0 | 1 | 2 | 6 | 5.144 | 2.343 | 1.969 | 7.822 | 9.505 | 14.006 | 4.666 | 3.984 | 50.395 |
| *Hypsiboas albomarginatus* | 1 | 1 | 1 | 3 | 6 | 1.905 | 1.402 | 1.060 | 3.870 | 5.099 | 7.187 | 1.750 | 2.299 | 21.582 |
| *Hypsiboas faber* | 1 | 0 | 1 | 3 | 6 | 8.337 | 4.265 | 3.312 | 11.908 | 13.475 | 20.339 | 6.325 | 6.299 | 63.804 |
| *Hypsiboas semilineatus* | 1 | 0 | 1 | 3 | 5 | 1.988 | 1.938 | 2.246 | 6.382 | 7.287 | 9.992 | 1.748 | 2.601 | 25.447 |
| *Itapotihyla langsdorffii* | 1 | 0 | 2 | 3 | 7 | 2.105 | 2.243 | 2.323 | 5.812 | 6.726 | 9.829 | 1.933 | 1.894 | 25.433 |
| *Leptodactylus latrans* | 1 | 0 | 1 | 2 | 5 | 4.756 | 3.721 | 3.130 | 9.349 | 10.795 | 18.631 | 4.835 | 2.705 | 56.729 |
| *Phyllomedusa distincta* | 2 | 0 | 2 | 2 | 5 | 1.073 | 0.358 | 0.562 | 2.020 | 1.981 | 2.605 | 0.656 | NA | 10.983 |
| *Physalaemus atlanticus* | 1 | 1 | 0 | 1 | 5 | 1.111 | 1.058 | 0.829 | 2.557 | 3.561 | 4.792 | 0.775 | 1.119 | 13.374 |
| *Physalaemus cuvieri* | 2 | 0 | 0 | 1 | 5 | 0.796 | 0.637 | 0.669 | 2.484 | 3.368 | 4.475 | 6.592 | 1.440 | 11.067 |
| *Rhinella ornata* | 1 | 1 | 1 | 1 | 5 | 0.695 | 0.717 | 0.701 | 2.270 | 2.640 | 3.885 | 0.445 | 1.158 | 9.445 |
| *Scinax* cf. *perereca* | 0 | 0 | 2 | 5 | 5 | 2.163 | 1.761 | 1.673 | 4.336 | 4.059 | 5.714 | 13.922 | 1.676 | 19.636 |
| *Scinax littoralis* | 2 | 0 | 1 | 2 | 5 | 0.707 | 0.615 | 0.449 | 1.463 | 2.104 | 3.076 | 5.943 | 0.755 | 9.019 |
| *Scinax trapicheiroi* | 1 | 0 | 1 | 2 | 5 | 1.347 | 1.089 | 1.005 | 2.338 | 2.986 | 4.278 | 2.224 | 1.170 | 13.288 |
| *Scinax alter* | 1 | 0 | 2 | 5 | 5 | 1.466 | 1.123 | 1.116 | 3.225 | 3.343 | 5.246 | 1.270 | 1.203 | 15.968 |
| *Scinax argyreornatus* | 0 | 0 | 1 | 3 | 5 | 0.595 | 0.526 | 0.452 | 1.386 | 1.908 | 2.970 | 0.495 | 0.608 | 8.041 |
| *Scinax* cf. *hayii* | 1 | 0 | 2 | 4 | 5 | 1.458 | 1.908 | 2.212 | 4.569 | 3.909 | 5.165 | 1.109 | 2.016 | 17.298 |
| *Trachycephalus mesophaeus* | 1 | 0 | 2 | 2 | 10 | 1.141 | 1.205 | 1.275 | 3.148 | 3.264 | 5.021 | 0.817 | 1.184 | 14.328 |
